# The genome sequence of the Six-striped Rustic, *Xestia sexstrigata *(Haworth, 1809)

**DOI:** 10.12688/wellcomeopenres.19988.1

**Published:** 2023-09-08

**Authors:** Douglas Boyes, John F. Mulley, University of Oxford and Wytham Woods Genome Acquisition Lab, Darwin Tree of Life Barcoding collective, Wellcome Sanger Institute Tree of Life programme, Wellcome Sanger Institute Scientific Operations: DNA Pipelines collective, Tree of Life Core Informatics collective, Darwin Tree of Life Consortium

**Affiliations:** 1 UK Centre for Ecology & Hydrology, Wallingford, England, UK; 2 Bangor University, Bangor, Wales, UK

**Keywords:** Xestia sexstrigata, Six-striped Rustic, genome sequence, chromosomal, Lepidoptera

## Abstract

We present a genome assembly from an individual female *Xestia sexstrigata* (the Six-striped Rustic; Arthropoda; Insecta; Lepidoptera; Noctuidae). The genome sequence is 638.3 megabases in span. Most of the assembly is scaffolded into 32 chromosomal pseudomolecules, including the W and Z sex chromosomes. The mitochondrial genome has also been assembled and is 15.36 kilobases in length. Gene annotation of this assembly on Ensembl identified 15,104 protein coding genes.

## Species taxonomy

Eukaryota; Metazoa; Eumetazoa; Bilateria; Protostomia; Ecdysozoa; Panarthropoda; Arthropoda; Mandibulata; Pancrustacea; Hexapoda; Insecta; Dicondylia; Pterygota; Neoptera; Endopterygota; Amphiesmenoptera; Lepidoptera; Glossata; Neolepidoptera; Heteroneura; Ditrysia; Obtectomera; Noctuoidea; Noctuidae; Noctuinae; Noctuini; *Xestia*; *Xestia sexstrigata* (Haworth, 1809) (NCBI:txid997545).

## Background

The Six-striped Rustic ( *Xestia sexstrigata*) is a noctuid moth, easily recognised by dark veins on an overall brownish background, with clearly-defined oval and kidney markings. Forewing length is around 15 to 17 mm, and adults readily come to light during the summer flight season, peaking in July-August in the UK. *X. sexstrigata* has a Palaearctic distribution, and is common across the UK ( NBN Atlas Partnership, 2023). There seems to be an association with damp habitats such as marches, fens, and damp woodlands ( Scalercio *et al.*, 2009; Waring *et al.*, 2017), and larvae feed on a variety of herbaceous plants, including hedge bedstraw, bramble, and ribwort plantain ( Henwood *et al.*, 2020). Larvae overwinter before pupating in late spring. *X. sexstrigata* larvae are indistinguishable from those of the Square-spot Rustic ( *Xestia xanthographa*) ( Henwood *et al.*, 2020), which can complicate field-based surveys ( Boyes *et al.*, 2021), and this genome sequence (and that of *X. xanthographa* ( Boyes *et al.*, 2022)) should prove useful in the identification of genomic regions that can discriminate the two using molecular methods. Although classified as “declining” in Great Britain in 2006 ( Conrad *et al.*, 2006), by 2019 this has been amended to “least concern” ( Fox *et al.*, 2019), and recent reports suggest a range expansion into southern and south-eastern Europe ( Nunes *et al.*, 2021; Rákosy & Rákosy, 2020).

*X. sexstrigata* was described in 1809 by Adrian Hardy Haworth in his ‘Lepidoptera Britannia’ as *Noctua 6-strigata*, which, in accordance with article 32.5.2.6 of The International Code of Zoological Nomenclature, had to become *sexstrigata*. *Xestia* is a large genus comprising many subgenera, and this genome assembly, together with those of other members of the genus, both completed (e.g. Boyes *et al.*, 2022; Broad *et al.*, 2022) and ongoing, will provide vital data for phylogenomic analyses and taxonomic revision of this group.

## Genome sequence report

**Figure 1.**
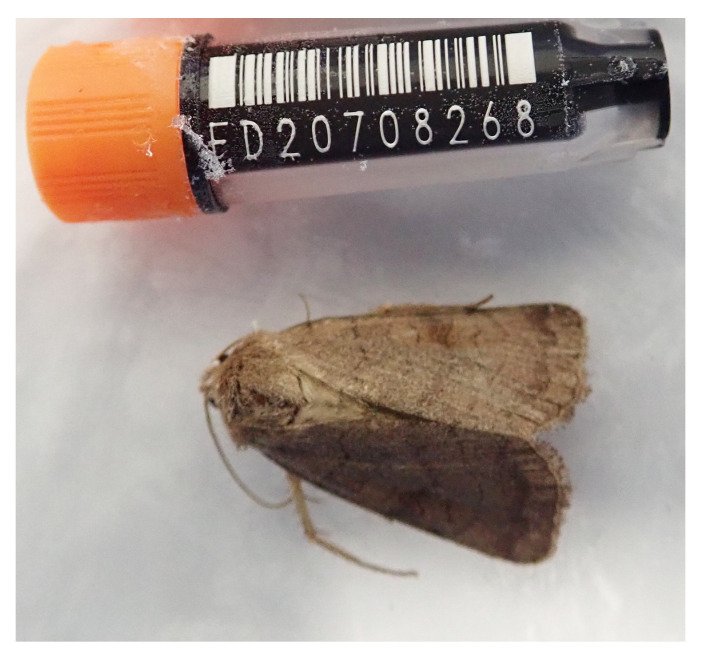
Photograph of the *Xestia sexstrigata* (ilXesSexs1) specimen used for genome sequencing.

The genome was sequenced from one female *Xestia sexstrigata* ( Figure 1) collected from Wytham Woods, Oxfordshire, UK (51.77, –1.34). A total of 29-fold coverage in Pacific Biosciences single-molecule HiFi long reads and 86-fold coverage in 10X Genomics read clouds were generated. Primary assembly contigs were scaffolded with chromosome conformation Hi-C data. Manual assembly curation corrected 32 missing joins or misjoins and removed 2 haplotypic duplications, reducing the scaffold number by 28.81%, and increasing the scaffold N50 by 0,54%.

**Figure 2.**
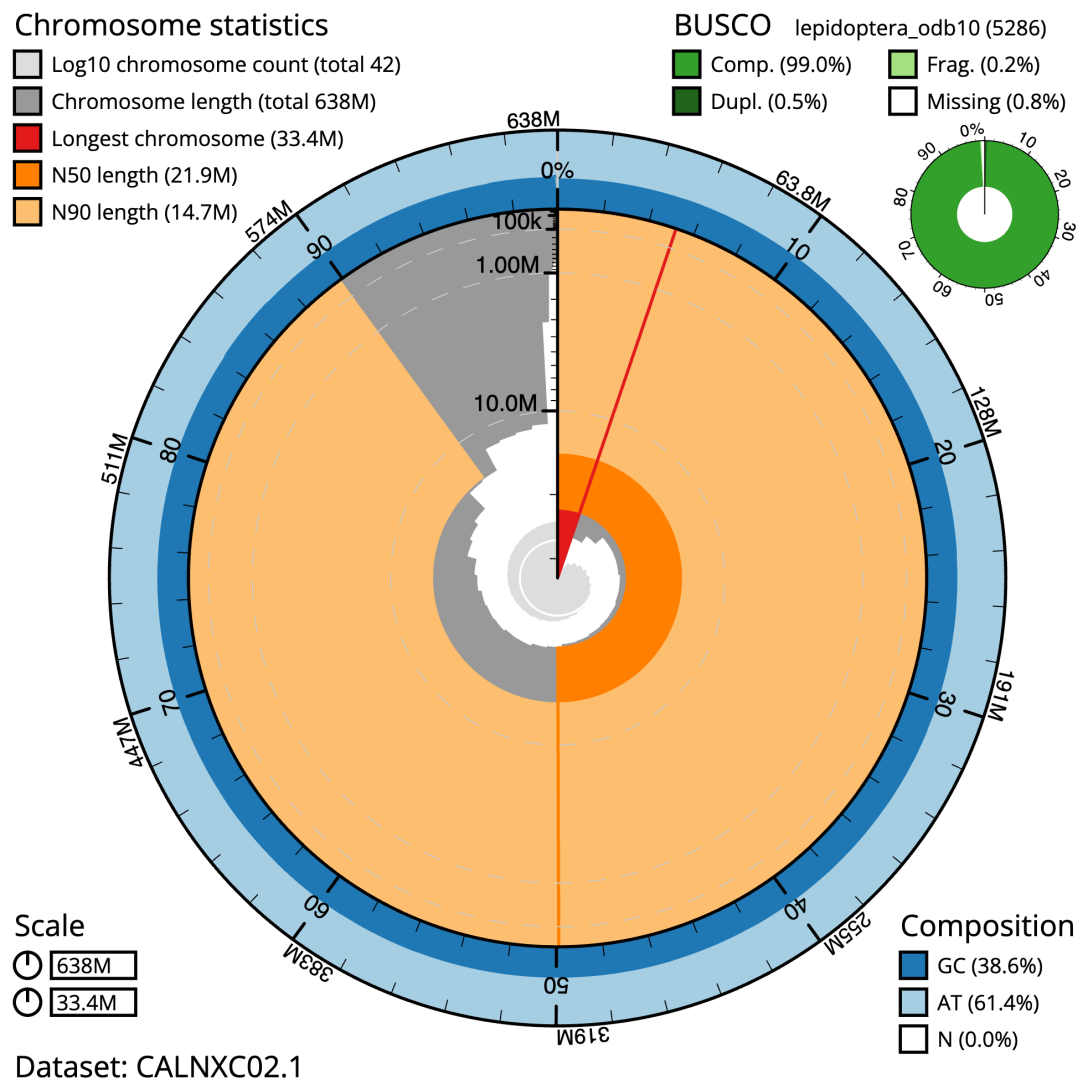
Genome assembly of *Xestia sexstrigata*, ilXesSexs1.2: metrics. The BlobToolKit Snailplot shows N50 metrics and BUSCO gene completeness. The main plot is divided into 1,000 size-ordered bins around the circumference with each bin representing 0.1% of the 638,333,203 bp assembly. The distribution of scaffold lengths is shown in dark grey with the plot radius scaled to the longest scaffold present in the assembly (33,407,853 bp, shown in red). Orange and pale-orange arcs show the N50 and N90 scaffold lengths (21,939,867 and 14,673,572 bp), respectively. The pale grey spiral shows the cumulative scaffold count on a log scale with white scale lines showing successive orders of magnitude. The blue and pale-blue area around the outside of the plot shows the distribution of GC, AT and N percentages in the same bins as the inner plot. A summary of complete, fragmented, duplicated and missing BUSCO genes in the lepidoptera_odb10 set is shown in the top right. An interactive version of this figure is available at https://blobtoolkit.genomehubs.org/view/ilXesSexs1.2/dataset/CALNXC02.1/snail.

The final assembly has a total length of 638.3 Mb in 41 sequence scaffolds with a scaffold N50 of 21.9 Mb ( Table 1). Most (99.98%) of the assembly sequence was assigned to 32 chromosomal-level scaffolds, representing 30 autosomes and the W and Z sex chromosomes. Chromosome-scale scaffolds confirmed by the Hi-C data are named in order of size ( Figure 2– Figure 5; Table 2). While not fully phased, the assembly deposited is of one haplotype. Contigs corresponding to the second haplotype have also been deposited. The mitochondrial genome was also assembled and can be found as a contig within the multifasta file of the genome submission.

**Table 1.** Genome data for *Xestia sexstrigata*, ilXesSexs1.2.

Project accession data		
Assembly identifier	ilXesSexs1.2	
Species	*Xestia sexstrigata*	
Specimen	ilXesSexs1	
NCBI taxonomy ID	997545	
BioProject	PRJEB52020	
BioSample ID	SAMEA8603196	
Isolate information	ilXesSexs1, female: thorax (DNA sequencing), head (Hi-C scaffolding), abdomen (RNA sequencing)	
Assembly metrics *		*Benchmark*
Consensus quality (QV)	66.4	≥ *50*
*k*-mer completeness	100%	≥ *95%*
BUSCO **	C:99.0%[S:98.5%,D:0.5%],F:0.2%,M:0.8%,n:5,286	*C* ≥ *95%*
Percentage of assembly mapped to chromosomes	99.98%	≥ *95%*
Sex chromosomes	W and Z chromosomes	*localised homologous pairs*
Organelles	Mitochondrial genome assembled	*complete single alleles*
Raw data accessions		
PacificBiosciences SEQUEL II	ERR9588938, ERR9588939	
10X Genomics Illumina	ERR9503450, ERR9503452, ERR9503453, ERR9503451	
Hi-C Illumina	ERR9503455	
PolyA RNA-Seq Illumina	ERR9503454	
Genome assembly		
Assembly accession	GCA_941918905.2	
*Accession of alternate haplotype*	GCA_941918865.2	
Span (Mb)	638.3	
Number of contigs	103	
Contig N50 length (Mb)	12.0	
Number of scaffolds	41	
Scaffold N50 length (Mb)	21.9	
Longest scaffold (Mb)	33.41	
Genome annotation of GCA_941918905.1		
Number of protein-coding genes	15,104	
Number of non-coding genes	4,043	
Number of gene transcripts	30,041	

* Assembly metric benchmarks are adapted from column VGP-2020 of “Table 1: Proposed standards and metrics for defining genome assembly quality” from ( Rhie *et al.*, 2021). ** BUSCO scores based on the lepidoptera_odb10 BUSCO set using v5.3.2. C = complete [S = single copy, D = duplicated], F = fragmented, M = missing, n = number of orthologues in comparison. A full set of BUSCO scores is available at https://blobtoolkit.genomehubs.org/view/ilXesSexs1.2/dataset/CALNXC02.1/busco.

**Figure 3.**
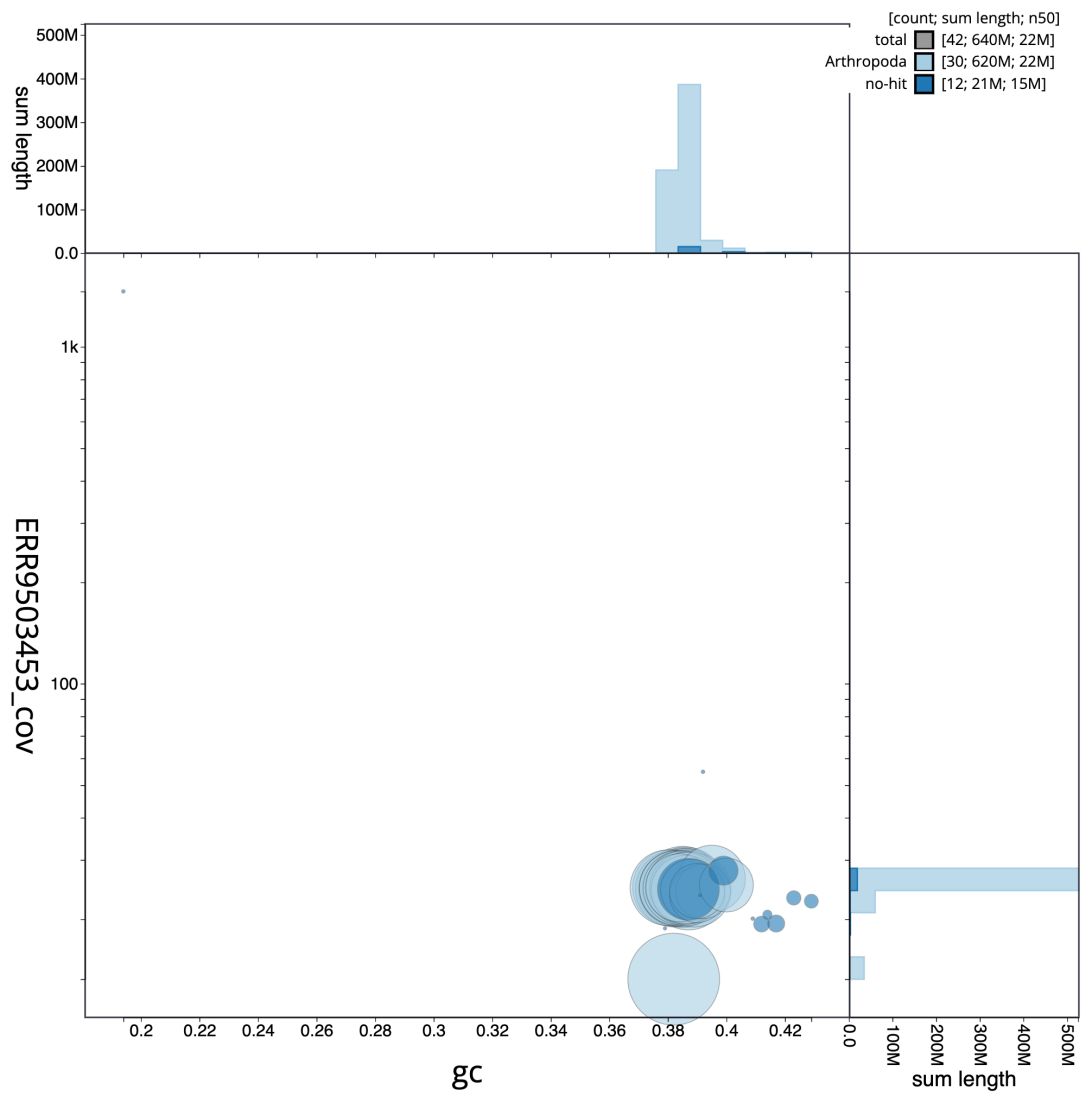
Genome assembly of *Xestia sexstrigata*, ilXesSexs1.2: BlobToolKit GC-coverage plot. Scaffolds are coloured by phylum. Circles are sized in proportion to scaffold length. Histograms show the distribution of scaffold length sum along each axis. An interactive version of this figure is available at https://blobtoolkit.genomehubs.org/view/ilXesSexs1.2/dataset/CALNXC02.1/blob.

**Figure 4.**
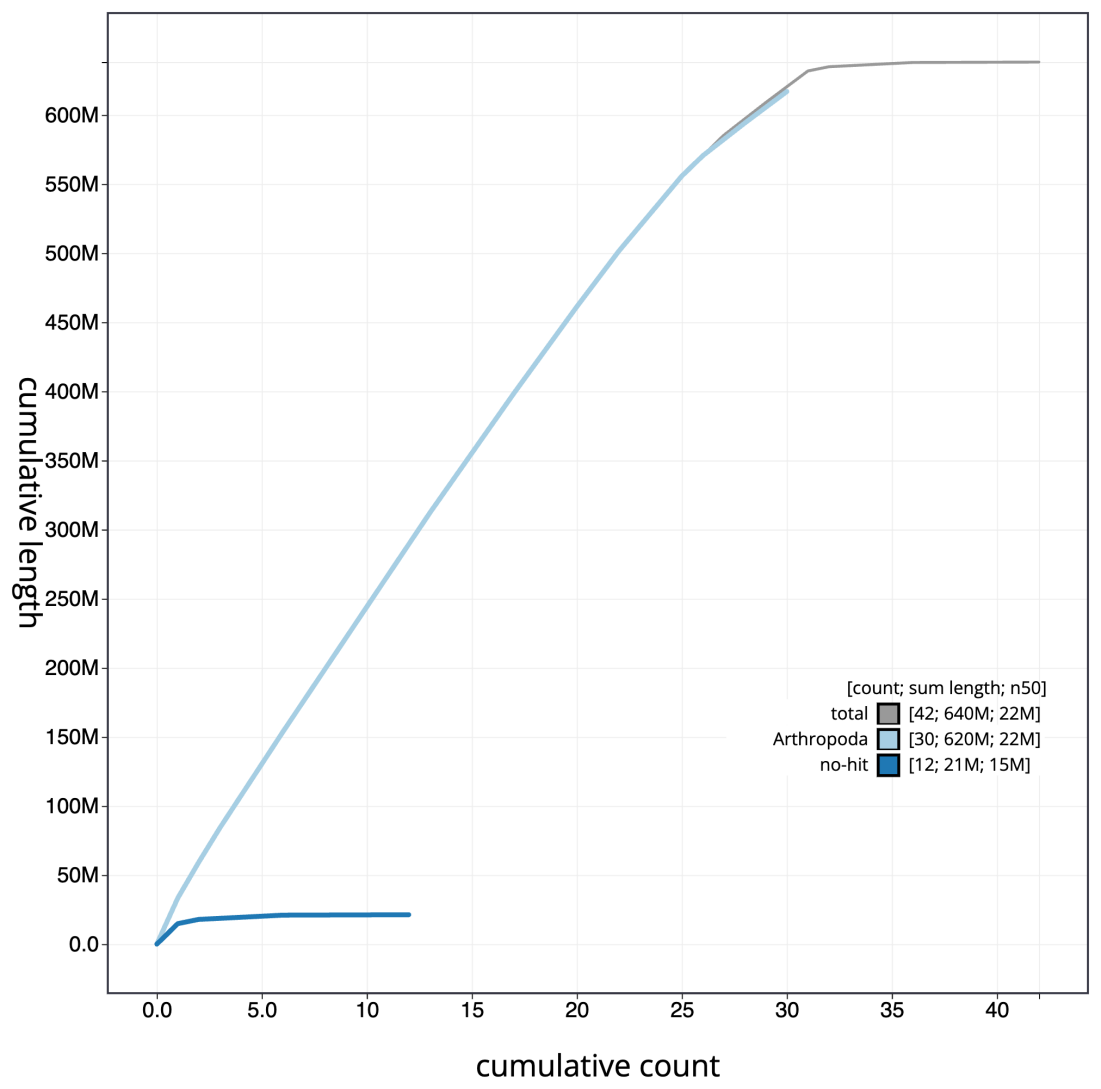
Genome assembly of *Xestia sexstrigata*, ilXesSexs1.2: BlobToolKit cumulative sequence plot. The grey line shows cumulative length for all scaffolds. Coloured lines show cumulative lengths of scaffolds assigned to each phylum using the buscogenes taxrule. An interactive version of this figure is available at https://blobtoolkit.genomehubs.org/view/ilXesSexs1.2/dataset/CALNXC02.1/cumulative.

**Figure 5.**
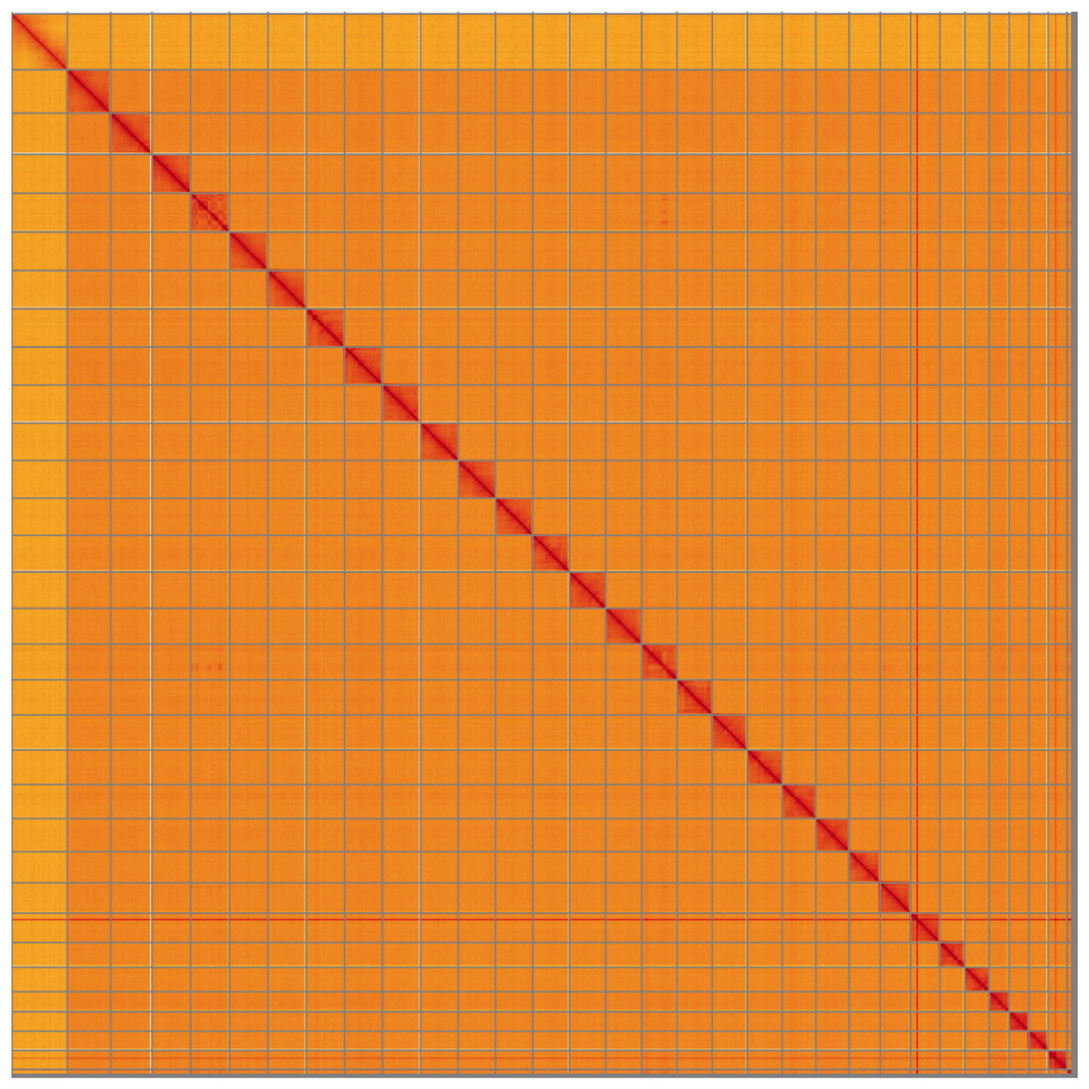
Genome assembly of *Xestia sexstrigata*, ilXesSexs1.2: Hi-C contact map of the ilXesSexs1.2 assembly, visualised using HiGlass. Chromosomes are shown in order of size from left to right and top to bottom. An interactive version of this figure may be viewed at https://genome-note-higlass.tol.sanger.ac.uk/l/?d=XkWYqodtQ4O9VQ6Ecw5pVw.

**Table 2.** Chromosomal pseudomolecules in the genome assembly of *Xestia sexstrigata*, ilXesSexs1.

INSDC accession	Chromosome	Length (Mb)	GC%
OW799201.2	1	25.98	38.5
OW799202.2	2	24.68	38.5
OW799204.2	4	23.19	38.5
OW799205.2	5	23.16	38.5
OW799203.2	3	23.15	38.5
OW799206.2	6	22.93	38.0
OW799208.2	8	22.91	38.0
OW799207.2	7	22.72	38.5
OW799211.2	11	22.69	38.5
OW799209.2	9	22.58	38.5
OW799212.2	12	22.41	38.0
OW799210.2	10	22.33	38.5
OW799214.2	14	21.94	38.5
OW799213.2	13	21.76	38.5
OW799216.2	16	21.4	38.5
OW799215.2	15	21.36	39.0
OW799217.2	17	21.06	38.5
OW799218.2	18	20.94	38.5
OW799219.2	19	20.77	38.5
OW799220.2	20	20.31	38.5
OW799221.2	21	19.91	38.5
OW799222.2	22	18.6	38.5
OW799223.2	23	18.18	38.5
OW799224.2	24	17.57	39.5
OW799225.2	25	14.84	38.5
OW799226.2	26	14.67	39.0
OW799227.2	27	11.93	39.0
OW799229.2	29	11.76	39.0
OW799228.2	28	11.57	39.0
OW799230.2	30	11.19	40.0
OW799231.2	W	3.07	40.0
OW799200.2	Z	33.41	38.0
OW799232.2	MT	0.02	19.5

The estimated Quality Value (QV) of the final assembly is 66.4 with *k*-mer completeness of 100%, and the assembly has a BUSCO v5.3.2 completeness of 99.0% (single = 98.5%, duplicated = 0.5%), using the lepidoptera_odb10 reference set ( *n* = 5,286).

Metadata for specimens, spectral estimates, sequencing runs, contaminants and pre-curation assembly statistics can be found at https://links.tol.sanger.ac.uk/species/997545.

## Genome annotation report

The *Xestia sexstrigata* genome assembly (GCA_941918905.1) was annotated using the Ensembl rapid annotation pipeline ( Table 1; https://rapid.ensembl.org/Xestia_sexstrigata_GCA_941918905.1/Info/Index). The resulting annotation includes 30,041 transcribed mRNAs from 15,104 protein-coding and 4,043 non-coding genes.

## Methods

### Sample acquisition and nucleic acid extraction

A female *Xestia sexstrigata* (specimen ID Ox000965, individual ilXesSexs1) was collected from Wytham Woods, Oxfordshire (biological vice-county Berkshire), UK (latitude 51.77, longitude –1.34) on 2020-09-08, using a light trap. The specimen was collected and identified by Douglas Boyes (University of Oxford) and snape-frozen on dry ice.

DNA was extracted at the Tree of Life laboratory, Wellcome Sanger Institute (WSI). The ilXesSexs1 sample was weighed and dissected on dry ice with tissue set aside for Hi-C and RNA sequencing. Thorax tissue was disrupted using a Nippi Powermasher fitted with a BioMasher pestle. High molecular weight (HMW) DNA was extracted using the Qiagen MagAttract HMW DNA extraction kit. Low molecular weight DNA was removed from a 20 ng aliquot of extracted DNA using the 0.8X AMpure XP purification kit prior to 10X Chromium sequencing; a minimum of 50 ng DNA was submitted for 10X sequencing. HMW DNA was sheared into an average fragment size of 12–20 kb in a Megaruptor 3 system with speed setting 30. Sheared DNA was purified by solid-phase reversible immobilisation using AMPure PB beads with a 1.8X ratio of beads to sample to remove the shorter fragments and concentrate the DNA sample. The concentration of the sheared and purified DNA was assessed using a Nanodrop spectrophotometer and Qubit Fluorometer and Qubit dsDNA High Sensitivity Assay kit. Fragment size distribution was evaluated by running the sample on the FemtoPulse system.

RNA was extracted from abdomen tissue of ilXesSexs1 in the Tree of Life Laboratory at the WSI using TRIzol, according to the manufacturer’s instructions. RNA was then eluted in 50 μl RNAse-free water and its concentration assessed using a Nanodrop spectrophotometer and Qubit Fluorometer using the Qubit RNA Broad-Range (BR) Assay kit. Analysis of the integrity of the RNA was done using Agilent RNA 6000 Pico Kit and Eukaryotic Total RNA assay.

### Sequencing

Pacific Biosciences HiFi circular consensus and 10X Genomics read cloud DNA sequencing libraries were constructed according to the manufacturers’ instructions. Poly(A) RNA-Seq libraries were constructed using the NEB Ultra II RNA Library Prep kit. DNA and RNA sequencing was performed by the Scientific Operations core at the WSI on Pacific Biosciences SEQUEL II (HiFi), Illumina HiSeq 4000 (RNA-Seq) and NovaSeq 6000 (10X) instruments. Hi-C data were also generated from head tissue of ilXesSexs1 using the Arima2 kit and sequenced on the Illumina NovaSeq 6000 instrument.

### Genome assembly, curation and evaluation

Assembly was carried out with Hifiasm ( Cheng *et al.*, 2021) and haplotypic duplication was identified and removed with purge_dups ( Guan *et al.*, 2020). One round of polishing was performed by aligning 10X Genomics read data to the assembly with Long Ranger ALIGN, calling variants with FreeBayes ( Garrison & Marth, 2012). The assembly was then scaffolded with Hi-C data ( Rao *et al.*, 2014) using YaHS ( Zhou *et al.*, 2023). The assembly was checked for contamination and corrected as described previously ( Howe *et al.*, 2021). Manual curation was performed using HiGlass ( Kerpedjiev *et al.*, 2018) and Pretext ( Harry, 2022). The mitochondrial genome was assembled using MitoHiFi ( Uliano-Silva *et al.*, 2023), which runs MitoFinder ( Allio *et al.*, 2020) or MITOS ( Bernt *et al.*, 2013) and uses these annotations to select the final mitochondrial contig and to ensure the general quality of the sequence.

A Hi-C map for the final assembly was produced using bwa-mem2 ( Vasimuddin *et al.*, 2019) in the Cooler file format ( Abdennur & Mirny, 2020). To assess the assembly metrics, the *k*-mer completeness and QV consensus quality values were calculated in Merqury ( Rhie *et al.*, 2020). This work was done using Nextflow ( Di Tommaso *et al.*, 2017) DSL2 pipelines “sanger-tol/readmapping” ( Surana *et al.*, 2023a) and “sanger-tol/genomenote” ( Surana *et al.*, 2023b). The genome was analysed within the BlobToolKit environment ( Challis *et al.*, 2020) and BUSCO scores ( Manni *et al.*, 2021; Simão *et al.*, 2015) were calculated.

Table 3 contains a list of relevant software tool versions and sources.

**Table 3.** Software tools: versions and sources.

Software tool	Version	Source
BlobToolKit	4.1.5	https://github.com/blobtoolkit/blobtoolkit
BUSCO	5.3.2	https://gitlab.com/ezlab/busco
FreeBayes	1.3.1-17-gaa2ace8	https://github.com/freebayes/freebayes
gEVAL	N/A	https://geval.org.uk/
Hifiasm	0.16.1	https://github.com/chhylp123/hifiasm
HiGlass	1.11.6	https://github.com/higlass/higlass
Long Ranger ALIGN	2.2.2	https://support.10xgenomics.com/genome-exome/software/pipelines/latest/advanced/other-pipelines
Merqury	MerquryFK	https://github.com/thegenemyers/MERQURY.FK
MitoHiFi	2	https://github.com/marcelauliano/MitoHiFi
PretextView	0.2	https://github.com/wtsi-hpag/PretextView
purge_dups	1.2.3	https://github.com/dfguan/purge_dups
sanger-tol/genomenote	v1.0	https://github.com/sanger-tol/genomenote
sanger-tol/readmapping	1.1.0	https://github.com/sanger-tol/readmapping/tree/1.1.0
YaHS	yahs-1.1.91eebc2	https://github.com/c-zhou/yahs

### Genome annotation

The Ensembl gene annotation system ( Aken *et al.*, 2016) was used to generate annotation for the *Xestia sexstrigata* assembly (GCA_941918905.2). Annotation was created primarily through alignment of transcriptomic data to the genome, with gap filling via protein-to-genome alignments of a select set of proteins from UniProt ( UniProt Consortium, 2019).

### Wellcome Sanger Institute – Legal and Governance

The materials that have contributed to this genome note have been supplied by a Darwin Tree of Life Partner. The submission of materials by a Darwin Tree of Life Partner is subject to the **‘Darwin Tree of Life Project Sampling Code of Practice’**, which can be found in full on the Darwin Tree of Life website here. By agreeing with and signing up to the Sampling Code of Practice, the Darwin Tree of Life Partner agrees they will meet the legal and ethical requirements and standards set out within this document in respect of all samples acquired for, and supplied to, the Darwin Tree of Life Project. 

Further, the Wellcome Sanger Institute employs a process whereby due diligence is carried out proportionate to the nature of the materials themselves, and the circumstances under which they have been/are to be collected and provided for use. The purpose of this is to address and mitigate any potential legal and/or ethical implications of receipt and use of the materials as part of the research project, and to ensure that in doing so we align with best practice wherever possible. The overarching areas of consideration are:

- Ethical review of provenance and sourcing of the material
- Legality of collection, transfer and use (national and international)

Each transfer of samples is further undertaken according to a Research Collaboration Agreement or Material Transfer Agreement entered into by the Darwin Tree of Life Partner, Genome Research Limited (operating as the Wellcome Sanger Institute), and in some circumstances other Darwin Tree of Life collaborators.

## Data Availability

European Nucleotide Archive: X *estia sexstrigata* (six-striped rustic). Accession number PRJEB52020; https://identifiers.org/ena.embl/PRJEB52020. ( Wellcome Sanger Institute, 2022) The genome sequence is released openly for reuse. The *Xestia sexstrigata* genome sequencing initiative is part of the Darwin Tree of Life (DToL) project. All raw sequence data and the assembly have been deposited in INSDC databases. Raw data and assembly accession identifiers are reported in Table 1.

## Author information

Members of the University of Oxford and Wytham Woods Genome Acquisition Lab are listed here: https://doi.org/10.5281/zenodo.4789928.

Members of the Darwin Tree of Life Barcoding collective are listed here: https://doi.org/10.5281/zenodo.4893703.

Members of the Wellcome Sanger Institute Tree of Life programme are listed here: https://doi.org/10.5281/zenodo.4783585.

Members of Wellcome Sanger Institute Scientific Operations: DNA Pipelines collective are listed here: https://doi.org/10.5281/zenodo.4790455.

Members of the Tree of Life Core Informatics collective are listed here: https://doi.org/10.5281/zenodo.5013541.

Members of the Darwin Tree of Life Consortium are listed here: https://doi.org/10.5281/zenodo.4783558.
