# Supplementary material for: The genome sequence of the Six-striped Rustic, Xestia sexstrigata (Haworth, 1809)
Source: Wellcome Open Res. 2023 Sep 8;8:399. [Version 1] doi: 10.12688/wellcomeopenres.19988.1 (PMC11109539; doi:10.12688/wellcomeopenres.19988.1)
Supplement: Peer review history

# Reviewer report: Reviewer response for version 1

**DOI:** 10.21956/wellcomeopenres.22134.r71103

**Published:** 2024-05-11

**Authors:** Hiroshi Arai

**Affiliations:** 1 Tokyo University of Agriculture and Technology, Tokyo, Japan

The authors constructed a very high-quality genome assembly from a single female Xestia sexstrigata. The assembled contigs were scaffolded into 32 chromosomes, including sex chromosomes. I think the genome data presented here will help further studies on biodiversity, pest management and insect evolution (such as sex chromosome evolution). This manuscript is well presented, and I have only a minor comment.

 1)  Have you cleaned up non-primary reads such as endosymbionts?

 Insects host many endosymbiotic microbes such as bacteria. The Darwin Tree of Life project has generated very high-quality genome data of insect endosymbionts such as Wolbachia (Vancaester, E., & Blaxter, M. (2023). Phylogenomic analysis of Wolbachia genomes from the Darwin Tree of Life biodiversity genomics project. PLoS Biology, 21(1), e3001972).

 You used DNA extracted from thorax of the insect for genome assembly, and I wonder if your sequence data contained gut microbes/endosymbionts. Did you check or discard this data before/after assembly? Did you find any homologies between the assembled contigs and known endosymbionts such as Wolbachia?

Are sufficient details of methods and materials provided to allow replication by others?

Yes

Is the rationale for creating the dataset(s) clearly described?

Yes

Are the datasets clearly presented in a useable and accessible format?

Yes

Are the protocols appropriate and is the work technically sound?

Yes

Reviewer Expertise:

Evolutionary biology, microbiology, entomology, and insect pathology. Genomic/transcriptomic analyses of insects and insect endosymbionts.

I confirm that I have read this submission and believe that I have an appropriate level of expertise to confirm that it is of an acceptable scientific standard.

# Reviewer report: Reviewer response for version 1

**DOI:** 10.21956/wellcomeopenres.22134.r73298

**Published:** 2024-05-11

**Authors:** Sivasankaran Kuppusamy

**Affiliations:** 1 Entomology Research Institute, Loyola Collège, Chennai, India

I congratulate the authors for assembling the whole genome sequence of Six-striped Rustic *Xestia sexstrigata*, (Haworth, 1809). The authors have employed genome assembly techniques, whole genome sequencing technology, and relevant software. Furthermore, they sequenced spanning 638.3 megabases in length for the study.

**Suggestions**

 Page No 2 Subheading “Background” the first sentence last line and last word Kidney markings. It can be changed as renal-shaped markings instead of Kidney markings.

Are sufficient details of methods and materials provided to allow replication by others?

Yes

Is the rationale for creating the dataset(s) clearly described?

Yes

Are the datasets clearly presented in a useable and accessible format?

Yes

Are the protocols appropriate and is the work technically sound?

Yes

Reviewer Expertise:

Phylogenetic analysis of Noctuoidea moths

I confirm that I have read this submission and believe that I have an appropriate level of expertise to confirm that it is of an acceptable scientific standard.

# Reviewer report: Reviewer response for version 1

**DOI:** 10.21956/wellcomeopenres.22134.r71100

**Published:** 2024-05-11

**Authors:** Axel Künstner

**Affiliations:** 1 University of Lübeck, Lübeck, Germany

Here, the authors present the genome sequence of the six-striped Rustic (X. sexstrigata). The data note is clearly written and covers all necessary points (rational, protocols, sufficient details on the methods).

 I would like to raise only one point. The methods section is well written and the reader can use the information given to re-do the provided assembly. However, I miss the options (run parameters) used for the individual programs/applications. Can you please add this information to the method section or to Table 3?

Are sufficient details of methods and materials provided to allow replication by others?

Partly

Is the rationale for creating the dataset(s) clearly described?

Yes

Are the datasets clearly presented in a useable and accessible format?

Yes

Are the protocols appropriate and is the work technically sound?

Yes

Reviewer Expertise:

Genomics, genome assembly, tumor landscape, microbiome, selection

I confirm that I have read this submission and believe that I have an appropriate level of expertise to confirm that it is of an acceptable scientific standard, however I have significant reservations, as outlined above.

# Reviewer report: Reviewer response for version 1

**DOI:** 10.21956/wellcomeopenres.22134.r71108

**Published:** 2024-02-20

**Authors:** Anandi Bierman

**Affiliations:** 1 Stellenbosch University, Stellenbosch, South Africa

The Genome sequence of the six-striped rustic, xestia sexstrigata presents a Lepidopteran genome from the Noctuidae family. The 638.3 Mbp genome produced with 32 chromosomal pseudomolecules - including sex chromosomes and mitochondrion is described. The genome is annotated with a total of 15,104 protein coding genes identified.

 The genome size and number of protein coding genes are in keeping with other lepidoptera in the genus Xestia, with genomes available on GenBank and Ensembl.

 The rationale of sequencing this organism is provided as providing information for phylogenomic analyses and taxonomic revision of the group, particularly in light of the other genome sequences available for this genus.

- The data is very clearly represented and particularly useful in its interactive formats, which are easy to access as was the genome sequence itself.
- The methods on DNA extraction and sequencing as well as sequence assembly are provided in sufficient detail that researchers with experience in genome sequencing and assembly could replicate the protocol.
- The methodology behind the annotation is lacking in sufficient detail to be replicable.
- The Ensembl rapid annotation pipeline seems good in terms of reproducibility but there is no clarity or explanation on the steps and software involved in this pipeline.
- The methods section briefly mentions that transcriptomic data was used and select proteins from the UniProt consortium - however no mention is made of how these proteins were selected? In addition, alignment to transcriptomic data was used for annotation, but no methodology is given on transcript profiling or identification of the transcriptomic data? Were transcriptomics reads aligned or was the transcriptome first assembled?
- More clarity or accessibility to the protocol for annotation is needed.

Specific comments per section:

 BACKGROUND

 Spelling: marches - marshes: "There seems to be an association with

 damp habitats such as marches, fens, and damp woodlands"

 GENOME SEQUENCE REPORT

 Figure 2: The snailplot generated with BlobToolkit is very informative and a good representation of the data. However, I find it very surprising that the GC content is so uniform across the genome? I did look at the interactive plot and see no major deviation from the 38% GC: 61% AT content. Is this simply a scale problem? please clarify.

 Figure 3: This figure is slightly difficult to understand and I am not sure if it adds any value in addition to the GC coverage shown by the snailplot. The plot only really made sense to me after searching for similar plots in other organisms, which put the patterns shown into context for me.

 METHODS

 Spelling: snape-frozen - snap-frozen. "The specimen was collected and identified by Douglas Boyes

 (University of Oxford) and snape-frozen on dry ice."

Are sufficient details of methods and materials provided to allow replication by others?

Partly

Is the rationale for creating the dataset(s) clearly described?

Yes

Are the datasets clearly presented in a useable and accessible format?

Yes

Are the protocols appropriate and is the work technically sound?

Partly

Reviewer Expertise:

Genomics, genetic markers, bioinformatics

I confirm that I have read this submission and believe that I have an appropriate level of expertise to confirm that it is of an acceptable scientific standard, however I have significant reservations, as outlined above.
